# Intraspinal anomalies in scoliosis: An MRI analysis of 177 consecutive scoliosis patients

**DOI:** 10.4103/0019-5413.58607

**Published:** 2010

**Authors:** S Rajasekaran, Vijay Kamath, R Kiran, Ajoy Prasad Shetty

**Affiliations:** Department of Orthopaedics and Spine Surgery, Ganga Hospital, 313, Mettupalayam Road, Coimbatore – 641 011, Tamil Nadu, India

**Keywords:** Intraspinal anomalies, MRI scan, neuro-axial anomalies, scoliosis

## Abstract

**Background:**

The association of intraspinal neural anomalies with scoliosis is known for more than six decades. However, there are no studies documenting the incidence of association of intraspinal anomalies in scoliotic patients in the Indian population. The guide lines to obtain an magnetic resonance imaging (MRI) scan to rule out neuro-axial abnormalities in presumed adolescent idiopathic scoliosis are also not clear. We conducted a prospective study (a) to document and analyze the incidence and types of intraspinal anomalies in different types of scoliosis in Indian patients. (b) to identify clinico-radiological ‘indicators’ that best predict the findings of neuro-axial abnormalities in patients with presumed adolescent idiopathic scoliosis, which will alert the physician to the possible presence of intraspinal anomalies and optimize the use of MRI in this sub group of patients.

**Materials and Methods:**

The data from 177 consecutive scoliotic patients aged less than 21 years were analyzed. Patients were categorized into three groups; Group A - congenital scoliosis (n=60), group B -presumed idiopathic scoliosis (n=94) and group C - scoliosis secondary to neurofibromatosis, neuromuscular and connective tissue disorders (n=23). The presence and type of anomaly in the MRI was correlated to patient symptoms, clinical signs and curve characteristics.

**Results:**

The incidence of intraspinal anomalies in congenital scoliosis was 35% (21/60), with tethered cord due to filum terminale being the commonest anomaly (10/21). Patients with multiple vertebral anomalies had the highest incidence (48%) of neural anomalies and isolated hemi vertebrae had none. In presumed ‘idiopathic’ scoliosis patients the incidence was higher (16%) than previously reported. Arnold Chiari-I malformation (AC-I) with syringomyelia was the most common neural anomaly (9/15) and the incidence was higher in the presence of neurological findings (100%), apical kyphosis (66.6%) and early onset scoliosis. Isolated lumbar curves had no anomalies. In group-C, incidence was 22% and most of the anomalies were in curves with connective tissue disorders.

**Conclusion:**

The high incidence of intraspinal anomalies in presumed idiopathic scoliosis in our study group emphasizes the need for detailed examination for subtle neurological signs that accompany neuro-axial anomalies. Preoperative MRI screening is recommended in patients with presumed ‘idiopathic’ scoliosis who present at young age, with neurological findings and in curves with apical thoracic kyphosis.

## INTRODUCTION

The vertebral column and spinal cord are closely related anatomically and developmentally, hence it is common to see an intraspinal anomaly associated with scoliosis. It has been noted that in congenital scoliosis, where the neural axis and the vertebral column are being formed in close relation to time and location, malformation in spinal column can be commonly associated with anomaly in the spinal cord, nerve roots or covering membranes.^1^

Although the etiology of “idiopathic” scoliosis is still unresolved, the association between “idiopathic” scoliosis and cranio-vertebral abnormalities such as syringomyelia or Chiari malformations has been well established. With the development of magnetic resonance imaging (MRI), neural axis abnormalities are increasingly being found in patients with presumed “idiopathic” scoliosis where scoliosis was the presenting sign of an otherwise asymptomatic neural axis abnormality. Although all of the detected anomalies may not require active intervention, pre-operative detection is important in patients who are undergoing manipulative correction as there is a risk of neurological deficits if they are not addressed prior to deformity correction.^2^–^5^ It is therefore important to investigate neural axis abnormalities prior to corrective surgery for scoliosis. MRI, being the most sensitive investigation at present to study the anatomy of the neural structures, will be the investigation of choice to detect these anomalies. The routine use of MRI remains controversial, and current indications for MRI in “idiopathic” scoliosis vary from study to study. Several clinico-radiological features (indicators) have been identified in these scoliotic patients who are known to be associated with a high incidence of such anomalies. Our aims were to (a) document the incidence of intraspinal anomalies in different types of scoliosis and (b) study the association of intraspinal anomalies with patient symptoms, clinical signs and curve characteristics to establish which of these indicators best predicts the finding of clinically relevant abnormality of the central nervous system in patients with presumed adolescent idiopathic scoliosis.

## MATERIALS AND METHODS

Between 2006 and 2008 a total of 198 patients with scoliosis were admitted to our institute for surgical management. Inclusion criteria for the study were: age of less than 21 years, all types of spontaneous onset scoliosis including kyphoscoliosis, patients with documented clinical and radiological findings with whole spine MRI done pre operatively. Exclusion criteria were: pure kyphosis, adult onset scoliosis, scoliosis secondary to bone destruction due to infection, tuberculosis and trauma; patients with clinically obvious spinal dysraphism. 177 patients fulfilled the criteria and were included in the final analysis.

The patients were divided on clinico-radiological features in to three broad groups according to the type of scoliosis as the prevalence and relation of intraspinal anomalies are known to be different in each type of scoliosis. Group-A (n=60) included congenital scoliosis patients, group-B (n=94), presumed idiopathic scoliosis and group-C (n=23) miscellaneous scoliosis including neuromuscular scoliosis, neurofibromatosis, and deformities due to connective tissue disorders. Presumed ‘idiopathic’ scoliosis included all patients who were diagnosed to have ‘idiopathic’ scoliosis at first presentation to the outpatient department.

We documented history, clinical findings and neurological examination, for every patient. Whole spine radiographs including bending views were evaluated for type of curve, side, extent, magnitude, sagittal alignment, flexibility, vertebral anomalies, bony spurs, and Riser's grade of skeletal maturity. Whole spine MRI scans was obtained for all patients and studied (by an experienced Radiologist) for any abnormalities in the hind brain, brainstem, spinal cord, nerve roots and the covering membranes. Arnold chiari-I malformation was considered when the tonsillar herniation was 5 mm or below the level of foramen magnum. Location and dimensions of the syringomyelia was noted if present and level of conus medullaris was documented. The presence and type of anomaly in the MRI was correlated to patient symptoms, clinical signs and curve characteristics which are identified as the ‘indicators’ risk factors for intraspinal anomalies.

### Statistical analysis

The patients in each group with intraspinal anomalies were compared to those without having associated intraspinal anomalies within the respective group with respect to clinical and radiological features of interest. Data were compared using chi square test for categorical values and unpaired T test for numerical values. The differences with a *P* value of <0.05 were considered as statistically significant.

## RESULTS

### Congenital scoliosis (Group-A)

In this group, 44 patients had multiple vertebral anomalies while 16 had single level hemivertebra. 35% (21 of 60) of patients with congenital scoliosis had intraspinal anomalies. The mean age of presentation was 5.2 years (9 months to 16 years). There were 41 females, of which 13 (32%) had intraspinal anomalies; among the 19 males, 8 (42%) had anomalies. The most common anomaly was tethered cord syndrome due to tight filum terminale which was seen in 10 patients.

Patients with multiple vertebral segment malformations had a 48% (21of 44) incidence of neural anomalies while none of the 16 patients with a single level hemi-vertebra had a neuraxial anomaly. There was no significant difference (*P*>0.05) in the incidence of intraspinal anomalies between right sided curves 34% (12 out of 35) and left sided curves 36% (9 out of 25). There was no correlation between the magnitude of scoliosis and the presence of intraspinal anomalies. The mean Cobb's angle was 45° (27-89°) in patients with intraspinal anomalies as compared to 47° (29-77°) in rest of the patients (*P*>0.05) [Table 1].

**Table 1:** Correlation of intraspinal anomalies and clinico-radiographic indicators in congenital scoliosis

Indicator	Number of patients	Incidence of intraspinal anomalies	Percentage
Neurological abnormalities	12	9	75
Cutaneous markers	6	4	66
Multiple vertebral anomalies:Single vertebral anomalies	44:16	21:0	48:0
Females: Males	41:19	13:8	32:42
Left: Right curves	25:35	09:12	36:34
Thoracic: Lumbar curves	54:6	19:02	35:33

Twelve (20%) of 60 patients had clinically detectable neurological abnomalities of which nine (75%) had intraspinal anomalies. Diastometamyelia was seen in three patients, tethered cord in three and both were present together in two patients; one child had an intra dural lipoma. Sixty-six per cent (four of six) patients with cutaneous markers had neuro-axial anomaly. All the 21 patients had surgical intervention for the intraspinal anomaly before correcting the spinal deformity. In the three patients who had neurological abnormalities (asymmetric abdominal reflex) but no intraspinal anomalies, the findings were attributed to stretching of the cord as these patients had large curves (all > 70°).

### Presumed idiopathic scoliosis (Group-B)

**Figure 1:**
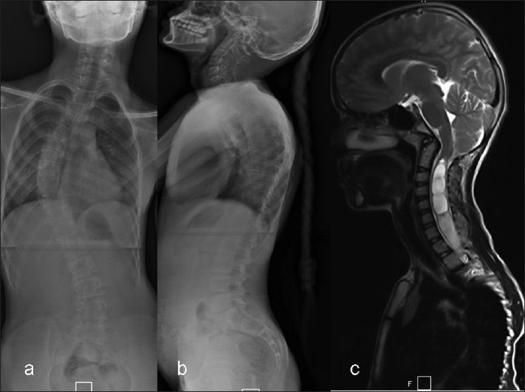
11 year old child with right thoracic presumed ‘idiopathic’ scoliosis. Clinically she had an asymmetrical abdominal reflex. (a, b) The radiographs showed a 53° right thoracic scoliosis with a thoracic kyphosis (T4-T12) of 38°. (c) The MRI showed a Arnold Chiari type-I malformation with a cervicothoracic syrinx. This required a foramen magnum decompression followed by posterior corrective instrumentation

This group consists of 94 patients who were classified as having “idiopathic” scoliosis at first presentation. These were sub divided into infantile (n=4), juvenile (n=11) and adolescent (n=79) scoliosis [Table 2]. The overall incidence of intra dural anomalies in this group was 16% (15/94). The most common intraspinal anomaly was syringomyelia associated with Arnold-Chiari malformation-I, (AC-I) seen in nine patients followed by four patients with isolated syringomyelia [Figure 1], one with AC-I malformation without syrinx and one patient had diplomyelia with a meningeal cyst. Syringomyelia was most commonly noted in the cervicothoracic region when it is associated with Arnold Chiari malformation.

**Table 2:** Summary of findings in presumed idiopathic scoliosis patients

Features	Number of patients	Number of intra-spinal anomalies	Incidence (%)
Adolescent scoliosis	79	11	14
Juvenile scoliosis	11	3	27
Infantile scoliosis	4	1	25
Subtle sensory motor Impairments	4	4	100
Abnormal abdominal reflexes	5	5	100
Apical thoracic kyphosis	9	6	66.6
Painful curves	5	1	20
Males	16	3	19
Females	78	12	16

The mean age of presentation was 11.4 (10-14) years in patients with intraspinal anomalies as compared to 13.2 (10-15) years in those who did not have and the difference was significant (*p*≤0.05). All patients presented with complaints of spinal deformity with five of them having pain, none had any neurological symptoms.

On clinical examination, nine (9.5%) of 94 patients showed subtle neurological impairment in the form of weakness and wasting of hand muscles, asymmetric loss of abdominal reflexes and extensor plantar reflex and all of them (100%) had an intraspinal anomaly. Thus the presence of a neurological impairment was an ‘indicator’ with a high yield for intraspinal anomalies on MRI scan.

**Figure 2:**
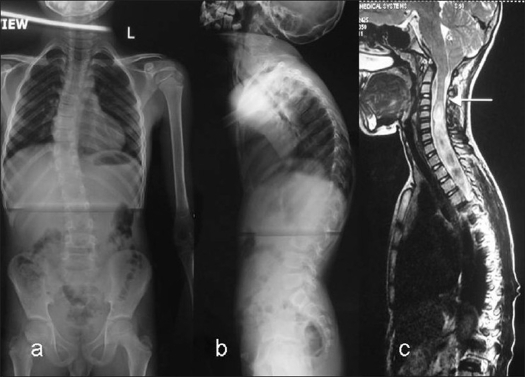
A 13 year old girl presented with a recent onset right thoracic scoliosis. Clinical examination was normal. (a, b) The whole spine X-ray showed a minimal coronal plane deformity, however there was a thoracic apical kyphosis. (c) The MRI showed a large cervico-thoracic syrinx

The mean coronal deformity was 47.4° (range 32° to 93°). The incidence of anomalies in curves greater than 60° was 18% (6 of 33) while in curves less than 60° it was 15% (9 of 61), however this difference was not significant (*P*≥0.05) [Table 3]. Patients with double curves (27%) had a significant higher incidence of intraspinal anomalies than those with single curves (10.7%) (*P*≤0.05) [Table 3]. There were 18 isolated lumbar curves which were left sided and none of these had any neuro-axial anomaly. The patients who had thoracic scoliosis with apical kyphosis [Figure 2] had a 66.6% incidence of intraspinal anomalies (six of nine patients).

Eleven (14%) of 79 patients with presumed adolescent “idiopathic' scoliosis had neuro-axial anomalies detected on MRI. Of these 11 cases, five had subtle neurological findings and three had atypical AIS curve patterns (apical kyphosis) while three (27.2%) patients had no abnormal clinical or radiography findings. Six (54.5%) of 11 patients with anomalies had normal neurology.

Seventy one patients satisfied all criteria to be classified as AIS (i.e. -a negative clinical history, a normal physical examination, and a typical adolescent idiopathic scoliosis curve pattern). Of these 71 AIS cases three (4.2%) had anomalies. All three patients had isolated syringomyelia.

All the patients with Arnold Chiari malformation were operated for foramen magnum decompression before scoliosis correction. Patients with isolated syringomyelia with normal neurology underwent only a scoliosis correction. None of them worsened neurologically after surgery. The infantile scoliosis patients were four in number out of which one patient had syringomyelia with no neurological deficits clinically. There were 11 juvenile scoliosis patients of which three had Arnold Chiari malformation with syringomyelia. All these patients had apical thoracic kyphosis. All the curves were in the thoracic region and nine were right sided and two were left sided. It was noticed that there is significantly higher incidence in these younger patients (27%) as compared to adolescent scoliosis patients (14%). As the sample size was very small, no statistical correlation could be established between the neural anomalies and the spinal deformity within each group in these patients.

The most valuable specific indicators i.e., those yielding the highest percentages of abnormal magnetic resonance images were - presence of neurological findings [associated with an abnormal finding in 9 (100%) of 9 patients], apical thoracic kyphosis [six (66.6%) of nine patients], double curves [eight (27%) of 29 patients] and early onset curves.

### Miscellaneous types of scoliosis (Group-C)

This group includes a total number of 23 patients out of which, seven patients had curves secondary to neurofibromatosis, ten patients were due to connective tissue disorders and six patients were due to neuromuscular etiology. The overall incidence of intraspinal anomalies in this group was 22% (5/23) most of which were seen in scoliosis secondary to connective tissue disorders (4/10). Syringomyelia was the most common anomaly which was present in all these patients, 4 in cervicothoracic junction and one in dorsal (T-9) region. Two of these patients additionally had AC-I malformation. The patients with AC-I malformation needed surgical treatment before deformity correction [Table 4].

**Table 3:** Incidence of intraspinal anomalies in different spinal curves in presumed idiopathic scoliosis patients

Feature	Number of patients	Number of intraspinal anomalies	Incidence (%)
Curve pattern			
Thoracic curves	47	7	15
Lumbar curves	18	Nil	Nil
Double curves	29	08	27
Type of thoracic curve			
Left sided thoracic curves	9	2	22
Right sided thoracic curves	67	13	19.5
Curve magnitude			
Curves < 60°	61	9	15
Curves > 60°	33	6	18

**Table 4:** Incidence of intraspinal anomalies in scoliosis due to neurofibromatosis, connective tissue and neuromuscular etiology

Feature	Neurofibromatosis (n=7)	Connective tissue disorder (n=10)	Neuromuscular (n=6) PPP -5 PTS -1
Males: Females	4:3	3:7	3:3
Right: Left sided curves	6:1	9:1	4:2
Single: Double curves	7:0	7:3	2:4
Curve magnitude	46° (32° to 83°)	47° (34° to 66°)	97° (67° to 122°)
Curve magnitude <60°: >60°	5:2	7:3	0:6
Neurocutaneous markers	7	Nil	Nil
Neurological deficits	0	3	6
Intraspinal anomalies	Syringomyelia - 1	ACM with syringomyelia – 2 syringomyelia - 2	None (post traumatic patient had cord signal changes)

PPPS - Post polio paralytic scoliosis, PTS – Post traumatic scoliosis, ACM – Arnold chiari malformation

Three of the 10 patients had subtle motor weakness in the hand muscles with absent abdominal reflex and all of them had an intraspinal anomaly. Patients with AC-I malformation underwent surgical decompression of foramen magnum before deformity correction. The mean Cobb's angle in patients with the neural anomalies was 47° (34-66°) which was similar to rest of the patients who did not have neural anomalies who had a mean angle of 49° (38-80°). Both the patients who had apical thoracic kyphosis had intraspinal anomalies detected on MRI screening. Incidentally, all patients with neural anomalies had single right thoracic scoliosis [Table 4].

Of the seven patients with scoliosis due to neurofibromatosis, one had a syringomyelia of the cervicothoracic region with no neurological deficits. There were six patients in the neuromuscular scoliosis group, five due to childhood poliomyelitis and one due to post traumatic paraplegia. All of them had severe coronal deformity of mean of 97° (67-122°), with associated neurological deficits. None of them had primary neural anomaly detected in the MRI. The patient with post traumatic paraplegia had myelomalacic changes in the cord secondary to trauma.

## DISCUSSION

The association of intraspinal anomalies with scoliosis is known since the last six decades but clear cut guidelines are lacking to identify and treat these conditions. MRI is the imaging modality of choice to identify these anomalies; however, the indications for MRI especially in presumed adolescent idiopathic scoliosis have not been clearly established. Guidelines to identify patients at “high risk” for neuro-axial anomalies need to be established. Documenting the incidence, type of anomaly and clinico-radiographic ‘indicators’ of scoliosis associated with a “high risk” for intraspinal anomalies will help the physician to judiciously choose patients requiring further investigation to rule out neuro-axial anomalies.

### Congenital scoliosis

In the current study, the incidence of intraspinal anomalies was 35%, which was within the range reported to vary from 18.3% to 38%;^1^,^6^–^7^ the high incidence had led to the general consensus that a preoperative MRI is essential in all patients before correction of a congenital scoliosis. Tethered cord syndrome due to tight filum terminale was the most common type of spinal anomaly noted in this study which was similar to observations reported in other studies.^8^ Patients with curves due to isolated hemi vertebrae showed no intraspinal anomalies in this study which was in contrast to observations of Philip *et al*.^9^ who reported equal incidence of intraspinal anomalies and similar rates of subsequent neurosurgical intervention in patients who have an isolated hemi vertebra and those who have a complex hemi vertebral pattern.

All the patients with neurological impairment had intraspinal anomaly making it the most sensitive clinical indicator. McMaster^1^ reported 4.8% incidence of neurological abnormalities with no detectable intraspinal anomalies on myelograms. MRI being a more sensitive investigation could detect the neural anomalies which could not be detected in myelogram. In the current study, all the patients with intraspinal anomalies associated with congenital scoliosis needed neurosurgical intervention before surgical correction of the deformity. The high risk indicators for a neuroaxial anomalies are presence of neurological abnormalities, multiple vertebral anomalies, presence of cutaneous markers and low risk indicator is isolated hemi vertebrae.

### Presumed idiopathic scoliosis

In the current study, the prevalence of abnormality of the central nervous system was approximately 16% in patients with presumed ‘idiopathic’ scoliosis. These patients presented with subtle abnormalities as identified on the basis of the clinical history, physical examination, or radiographic examination. Inoue *et al*.^10^ in a similar study reported an incidence of 18% in presumed ‘idiopathic’ scoliosis. This is significantly higher than the two to four per cent reported in prospective studies in adolescent idiopathic scoliotic (AIS) patients.^11^–^13^ On further analysis of our cohort of AIS (n=71) we had an incidence of 4.2% of neuro-axial abnormalities. The incidence was 27% in juvenile idiopathic scoliosis patients and 25 % in infantile scoliosis patients which is in the range reported in the literature.^14^–^17^

The mean age of onset of deformity was 11.4 years in those who had intraspinal anomalies as compared to the AIS group which was 13.2 years indicating that early age of onset is an indicator for presence of such anomalies. There is a significant correlation between age of onset of deformity and intraspinal anomaly in idiopathic group of patients (*P*≤0.05). This was similar to observations made in a prior study^18^ which found the mean age of patients with intraspinal anomalies lower (12 years) as compared to AIS patients (13 years).

No correlation was observed between the curve magnitude and incidence of neural anomalies but double curves had higher incidence than single curves. In the current study, none of the 18 isolated lumbar idiopathic curves had intraspinal anomalies.

Left thoracic curves have been associated with a high incidence (up to 50%) of syringomyelia^9^,^18^–^22^ and it has been used as an indicator of the presence of intraspinal anomalies in presumed ‘idiopathic’ scoliosis. In our series however there was no significant difference in the incidence of intraspinal anomalies between right and left sided curves, also on further analysis of the three AIS patients with left thoracic curves, none had an intraspinal anomalies. The lack of this association has been reported in other studies as well.^3^,^22^–^23^

The absence of a thoracic apical segment lordosis or the presence of thoracic apical kyphosis has been consistently associated (up to 80%^9^) with neuro-axial abnormalities in idiopathic scoliosis.^18^–^19^,^24^–^26^ In the current study, 66.6% (6 of 9) patients who had apical thoracic kyphosis had neuro-axial anomalies making it an important marker for detection intraspinal anomalies. All the nine patients with subtle neurological abnormalities were positive for intraspinal anomalies. This was in contrast to the reports of Davids *et al*,^27^ who reported only six per cent incidence of neural anomalies in patients with neurological abnormalities alone as risk factor.

Of interest in our study was the finding that 54.5% of patients with neuro-axial anomalies had no neurological abnormalities. It has been reported that up to 60% patients with scoliosis secondary to syringomyelia and 56% children with Chiari malformations had no detectable neurologic deficits.^28^–^29^ Also of note was that 27.2% of patients with neur-axial anomalies were clinically and radiographically normal. This implies that one in three AIS patients with neur-axial anomalies may get operated upon without detection if the presently used criteria for ordering an MRI are used. This is of concern as the risk of neurological injury during instrumented correction of scoliosis without prior decompression of an associated syrinx is known.^2^–^5^,^23^

The clinical significance of syringomyelia or Chiari malformations without accompanying neurologic deficits is not fully understood. In our study, however, the three patients who had normal clinical and radiological features, but with neuro-axial anomalies, underwent arthrodesis without any neurosurgical intervention, and post operatively were normal. They all had isolated syringomyelia. Though the numbers are too small to derive any unequivocal conclusions we can theorise that intraspinal anomalies without any abnormal clinical and radiographic features are probably minor enough not to require any surgical intervention and hence are clinically not relevant. Mashathoshi *et al*.^10^ in their series of presumed adolescent ‘idiopathic’ scoliosis had sixteen neurologically asymptomatic patients with abnormal findings on MRI; none of these patients had an indication for surgery, and no patient developed any permanent neurologic complication as a result of scoliosis surgery. They suggested that neurologically normal patients with “adolescent” idiopathic scoliosis are not indicated for routine preoperative MRI study and also those patients with “idiopathic” scoliosis, whose neurologic status is normal, might entail little risk of neurologic complications as a result of scoliosis surgery even if these patients have a neural axis malformation on MRI.

The most valuable single indicator of abnormality of the central nervous system as determined with MRI in the study group was presence of neurological deficit (100%) followed by the presence of apical thoracic segment kyphosis (66.6%) on the lateral plain radiograph of the spine. High risk indicators are presence of neurological deficits, apical thoracic kyphosis, double curves, early age of onset and low risk indicators are lumbar curves.

The current guidelines for MRI screening in scoliosis are valuable, and the proposed indications for ordering MRI in the literature include neurologic deficits,^18^ infantile and juvenile onset,^14^,^15^,^17^ male gender^30^, abnormal sagittal profile of the spine,^18^,^19^,^24^–^26^ atypical curve pattern (left-sided curve),^9^–^18^ rapid curve progression^27^ and the presence of pain.^18^,^27^ Our study so far recommends that indications for MRI analysis of neural axis abnormalities in presumed ‘idiopathic’ scoliosis should include early age of onset (the age at first presentation < 11 years), apical thoracic kyphosis, double curves and neurologic deficits including asymmetric superficial abdominal refiexes.

One of the drawbacks of our study is the small number of patients. Hence a study with larger data is suggested. In our study we could not stratify the usefulness of curve progression as an indicator of neuro-axial anomalies.

### Miscellaneous scoliosis

There appears to be higher incidence of neural anomalies in patients with secondary scoliosis due to connective tissue disorders and neurofibromatosis who have atypical curve pattern commonly and have rapid progression. The incidence of these anomalies in such patients was 22% in the current study. Therefore it is safe to consider these patients for preoperative MRI to identify and manage them before curve correction.

## CONCLUSION

The incidence of intraspinal anomalies in presumed ‘idiopathic’ scoliosis is high (16%). On the basis of this study, we believe that preoperative MRI is necessary for a patient with presumed ‘idiopathic’ scoliosis with neurological deficits, early onset (infantile and juvenile) of scoliosis, double curves and apical thoracic kyphosis. We stress the importance of a thorough history and physical evaluation, detailed neurologic examination, for patients with presumed ‘idiopathic’ scoliosis to document findings as subtle as an asymmetric superficial abdominal reflex.

The incidence of intraspinal anomalies in congenital scoliosis patients is 35% so the preoperative MRI is necessary to rule out intraspinal anomalies prior to surgical intervention.
